# The need to better understand aging and risk factors for dementia

**DOI:** 10.3389/frdem.2023.1346281

**Published:** 2024-01-08

**Authors:** Zoe Arvanitakis

**Affiliations:** Rush Alzheimer's Disease Center, Rush University, Chicago, IL, United States

**Keywords:** aging, dementia, Alzheimer's disease, risk factor, protective factor, modifiable

## Introduction

The biomedical journal *Frontiers in Dementia* provides a new and needed platform to showcase cutting-edge research in the common and increasingly impactful health condition of dementia. And as for many health conditions, prevention is a more attractive goal than treatment once a disease has already begun. With this lens, the “Aging and Risk Factors for Dementia” section of the journal covers etiological factors, with a focus on environmental factors, that instigate pathogenic mechanisms. The section publishes high-quality translational and clinical biomedical human studies, primarily about dementia risk factors including those that can be modified. Fields of focus in this section include the effects of aging and a range of risk and protective factors, on the manifestations of neurocognitive dysfunction and related conditions, and the neurobiologic underpinnings linking age and risk factors to dementia. Such fundamental topics are critical to advance scientific knowledge about dementia and to ultimately have an impact on clinical care and improved public health for persons with, and at risk for dementia.

## Why study aging and dementia?

Population trends around the globe have demonstrated that the average life expectancy is trending higher than in prior decades, and that the proportion of older persons in populations is increasing. Along with these trends, diseases of aging are becoming increasingly common and negatively impacting individuals, families, communities, and societies as a whole, more than ever. More specifically, the global societal burden of dementia is already very high, with 55 million persons affected by dementia, and with a worldwide cost of US $1.3 trillion (Alzheimer's Disease International, 2023a). Further, the dementia impact is becoming increasingly higher over time as populations continue to age. Indeed, age is considered one of the most important risk factors for dementia. While not all risk factors are recognized and while many existing risk factors need more study, a better understanding of risk factors offers the possibility for faster and greater improvements in patient clinical care and societal public health.

The public health challenge posed by aging diseases such as dementia may be mitigated by research on the causes of disease. As our research group and others have now shown, the most common cause of dementia is mixed etiology dementia, which most often encompasses a combination in the same individual, of a neurodegenerative process and usually Alzheimer's disease (AD), with a cerebrovascular disease and often brain infarcts. Many other combinations of two or more pathologies have been recognized. These brain pathologies tend to gradually accumulate in the brain and then once a threshold is reached, manifest initially as subtle cognitive changes and eventually as a dementia syndrome. Dementia usually presents as a gradual onset and progressive condition, accompanied by significant disability over the years until death. AD is a common, chronic, disabling disease, and the 7th leading cause of death in the US. AD dementia currently affects about 6.7 million people in the USA and is expected to affect 14 million by 2060, if no better treatments are introduced (Centers for Disease Control and Prevention, 2023). Vascular contributions to Cognitive Impairment and Dementia (VCID) is also very common and associated with disability and death, but less is known about the prevalence and other features of this condition. Yet other medical conditions have been found to affect the brain deleteriously, whether directly or indirectly, and may further contribute to chronic cognitive impairment, such as seen in some cases with “long COVID” resulting from the recent world-wide pandemic of COVID-19 (Adjaye-Gbewonyo et al., 2023). As a whole, Alzheimer's Disease/Alzheimer's Disease-Related Dementias (AD/ADRD) are complex disorders which clinicians are increasingly well-equipped to diagnose and treat (Arvanitakis et al., 2019), but for which there is currently no cure or good treatment without significant risks or barriers. Thus, underlying pathobiologic mechanisms in AD/ADRD, including as related to risk factors for aging and dementia, urgently need further elucidation to improve diagnosis, treatment, and prevention, and decrease global societal burden. Now is the time to elucidate these mechanisms, through research published in this and other journals, in order to address the enormous public health challenge presented by dementia.

## What are risk factors for dementia?

In the health care realm, a risk factor has been defined as “any attribute, characteristic or exposure of an individual that increases the likelihood of developing a disease or incurring an injury” (Organisation for Economic Co-operation and Development, 2023). There are several approaches to classify risk factors, such as the often-used classification according to the effect on disease, whether the factor increases or decreases risk. More precisely, *risk* factors are “characteristics at the biological, psychological, family, community, or cultural level that precede and are associated with a higher likelihood of negative outcomes,” while *protective* factors are “characteristics associated with a lower likelihood of negative outcomes or that reduce a risk factor's impact” (Substance Abuse Mental Health Services and Administration, 2019). With this approach, protective factors can be considered positive or even “beneficial” countering factors. Other classifications of risk and protective factors include by timing of effect, or by likelihood that the factor can be modified (e.g., modifiable such as cardiovascular risk factors, or not, such as age). The Table 1 offers examples of putative risk and protective factors for dementia, classified by the likelihood that a factor is modifiable, understanding that research in this area is rapidly evolving. Indeed, new knowledge continues to accumulate on risk and protective factors, and science advancements allow impacts such that what may not be modifiable today, may be modifiable in the future. Currently, leaders in the field have suggested that modifying 12 risk factors may decrease the likelihood of dementia by up to 40%, through prevention or a delay of the onset of symptoms (Livingston et al., 2020). Many researchers and clinicians in the field believe that risk factors play a critical role in helping address the public health crisis of dementia.

**Table 1:** Risk and protective factors^*^ for dementia, according to likelihood that factor is modifiable.

		**Likelihood of factor being modifiable**	
		**Lower likelihood of being modifiable: non-modifiable factors**	**Higher likelihood of being modifiable: modifiable factors**
Effect of factor on dementia risk	Increases dementia risk: risk factor	Increasing biologic age Female biologic sex Genetic factors (e.g., APOE ε4) Genetic diseases (e.g., Down's syndrome) Personality traits (e.g., neuroticism)	Hypertension Insulin resistance, hyperglycemia, and diabetes Obesity in midlife and decreasing BMI in late-life Traumatic brain injury Infrequent or disrupted sleep Hearing loss Excessive alcohol Smoking Air pollution Depression Psychosocial stress (e.g., violence)
	Decreases dementia risk: protective factor	Genetic factors (family history of longevity with preserved health)	Higher level and better-quality education Increased cognitive activities Increased socialization Psychosocial characteristics (e.g., purpose in life) Healthy diet (e.g., MIND diet) Increased and diverse physical activities Built environment (e.g., ease of transportation to place of work) Exposure to natural environments

^*^While several dementia risk factors are established (e.g., increasing age), other factors are only putative at this time, and research is ongoing in this field. BMI, body mass index; MIND diet, Mediterranean-DASH Intervention for Neurodegenerative Delay diet.

Researchers have made advances in our understanding of individual dementia risk factors. Increasing age is thought to be the most important risk factor for dementia in old age (Alzheimer's Disease International, 2023b). It is estimated that almost half of persons living to be 80 years of age will have a dementia, and many persons especially in industrialized countries, are living that long. Yet, numerous other factors other than age have been identified as risks. We know that genetic risk factors are important, most notably the *Apolipoprotein E* ε*4* (APOE ε4) allele for late-onset dementia, but also rare autosomal dominant mutations for young-onset dementia (e.g., *presenilin 1* mutations). And other risk factors have been elucidated, such as specific medical conditions (e.g., diabetes, hypertension, others), social and behavioral factors (e.g., social isolation, limited physical activity, poor diet and nutrition), and environmental conditions (e.g., inadequate built environment, pollution, and others). And, several risk factors seem to map to particular time periods across a life course, from early-life, to mid-life, and to late-life (Livingston et al., 2020). Indeed, while education is considered an early-life risk factor, traumatic brain injury may confer most risk during mid-life, and chronic medical conditions such as diabetes may have most impact during late-life. As research in the field evolves, many investigators are focusing on *potentially modifiable* risk factors, with the goal of advancing scientific knowledge including via interventional clinical trials, and of ultimately improving clinical care and population health.

## What are emerging areas in the research of aging and dementia risk factors?

Scientists have studied risk and protective factors for disease over the centuries, but recent scientific discoveries and advanced technologies offer unprecedented opportunities to make rapid and significant impacts, including in the field of dementia. Emerging areas of research on aging and risk factors for dementia span what/who considerations (e.g., which population to study), and when (e.g., which time point along the life course), how (e.g., which discipline, approach, tool to use), and by whom (e.g., which research team) to conduct the study. First, the “what/who” to study shapes the scope of the research, from studies in the cell dish to animal models to humans, whether in a controlled setting (e.g., clinical trials) or the “natural/real world” setting (e.g., population studies). And within specific categories of what/who to study, research on subgroups will shed light on topical areas such as precision medicine and health disparities. For instance, the study of diverse populations is more relevant than ever, given the increased incidence of dementia in minority populations including African Americans, Native Americans, and Latinos. Further, modifiable risk factors such as vascular factors (e.g., diabetes) and exposures (e.g., alcohol) are higher in some of these populations, further compounding the risk. At the global level, diverse populations need to be included in research as dementia is increasing disproportionately in the low and middle-income countries, which are also less well equipped to address the mounting public health problems, compared to the high-income countries. Second, “when” to conduct the study will affect what is learnt. As outlined by the recent Lancet Commission, researchers are aiming to take a life course approach to comprehensively research the effects of age from in utero to death, and considering a range of manifestations of disease (from pre-clinical, to subtle features, to clinical expression from mild to advanced stages—including cognitive, motor, mood, behavior, and others), and a range of risk/protective factors across life (Livingston et al., 2020).

Next, “how” to conduct the research allows to apply novel study designs which are emerging, including those that leverage advanced approaches and across disciplines. For example, dementia risk factors studies can focus on biologic and/or exposome factors, or combinations of these, such as for specific medical, genetic, epigenetic, omic, psychological, environmental, social, exposome, and a myriad of other factors. Furthermore, technologic tools are evolving, and contribute to increased knowledge about the neurobiologic underpinnings linking age and risk factors to dementia, and about biomarkers of disease (e.g., in biofluids, with neuroimaging such as PET scans, etc.). In particular, blood biomarkers of AD are extensively researched and promise to improve clinical trials and move into clinical practice soon, with the hope of informing on diagnosis, prediction, and monitoring treatment response. Separately, innovative and robust analytic approaches in the study of aging and risk factors in dementia include state of the science analyses and big data applications (e.g., for omics, neuroimaging, etc.), for instance with artificial intelligence, machine learning, bioinformatics, and computational science.

Lastly, dementia research is evolving by considering “by whom” the research is being done and what resources are involved. Typically, research discoveries are accelerated by using a team approach, especially with multi- and inter-disciplinary teams. While regulatory and administrative aspects of research are mounting, care needs to be given to create a collaborative, transparent, and open science environment. Furthermore, adequate and varied resources need to be made available to support the research enterprise, with attention to deploy limited resources in proportion to the impact of the public health conditions being addressed. Given the significant amount of the economic resources (e.g., gross domestic product) directed toward healthcare and chronic diseases of aging and dementia in particular, public funding and infrastructure from federal, state, and local governments are needed. In the US and since 2012, there is a National Plan to Address Alzheimer's disease [National Plan to Address Alzheimer's Disease (NAPA), 2023]. In addition to public funds, private sectors whether for profit (e.g., industry) or not (non-for profits) are beginning to join efforts with other stakeholders, from patients and advocacy groups, to researchers, to public and private organizations (Milne, 2023). Working together, research is more likely and rapidly going to impact clinical care and public health, for persons and societies with, and at risk for dementia.

## Conclusions

In conclusion, despite recent advances in therapeutics for dementia including with the first disease modifying drugs that are approved by the US Food and Drugs Administration Agency (FDA), these drugs are not currently scalable to large populations, and the need to study aging and risk factors for dementia is just as pressing as ever. The journal *Frontiers in Dementia* is well poised to highlight this research and help disseminate biomedical knowledge widely. We now know a lot more about risk factors for dementia, and by focusing on modifiable factors, we can pave the way toward having more impactful and rapid outcomes in clinical care and global public health. Emerging areas of research include considerations of what/who, when, how, and by whom to conduct the research. Among these considerations, four dementia research areas are perhaps in most immediate need: (1) studying diverse populations; (2) taking a comprehensive approach to a wide range of risk and protective factors, and across the life course; (3) using robust study designs, and sophisticated tools and analyses; and (4) fostering stronger national and international collaborative teams, supported by governmental and non-governmental entities. This is certainly a time of hope in the field of aging and dementia risk and protective factors.

## Author contributions

ZA: Conceptualization, investigation, writing—original draft and review & editing.
